# Co-designing a cancer care intervention: reflections of participants and a doctoral researcher on roles and contributions

**DOI:** 10.1186/s40900-022-00373-7

**Published:** 2022-08-02

**Authors:** Mary Anne Lagmay Tanay, Jo Armes, Catherine Oakley, Lesley Sage, Deb Tanner, Jose Roca, Liz Bryson, Barbara Greenall, Lauren Urwin, Toni Wyatt, Glenn Robert

**Affiliations:** 1 Florence Nightingale Faculty of Nursing, Midwifery and Palliative Care, King’s College London, London, UK; 2 School of Health Sciences, Faculty of Health and Medical Sciences, University of Surrey, Guildford, UK; 3 Guy’s and St. Thomas’ NHS Foundation Trust, London, UK; 4 Patient Expert, London, UK; 5 Oncology and Haematology Rehabilitation Unit, Guy’s and St. Thomas’ NHS Foundation Trust, London, UK

**Keywords:** Experience-based co-design, Co-production, Patient and public involvement, Intervention development, Co-design, PPI, Cancer

## Abstract

**Background:**

Patient and Public Involvement is most usually framed in the context of designing, conducting and/or disseminating research. Participatory methods such as Experience-Based Co-Design (EBCD) further allow service users to directly engage in developing, testing and implementing interventions and services alongside healthcare staff. This paper aims to explore how participants in an EBCD project came—over time—to perceive their role and involvement in co-designing a cancer care intervention.

**Methods:**

The findings are based on our reflections, a research diary, email correspondence and fieldnotes from co-design events. Co-design participants who attended most of the ten co-design events took part through written reflections or audio-recorded video calls. Ten reflective pieces were collected from clinicians (n = 4), PPI group members/patient participants (n = 4), a doctoral researcher (n = 1) and a visual illustrator (n = 1). Inductive data analysis of participant reflections was carried out using reflexive thematic analysis. Meeting fieldnotes, email correspondence and the researcher’s diary were deductively analysed using the initial themes generated from this inductive analysis.

**Results:**

Five main themes were identified: (1) changing perception of roles during the co-design process, (2) defining a ‘co-designer’, (3) engagement and ownership, (4) role of the research facilitator in maintaining momentum, and (5) perceived benefits of involvement.

**Conclusion:**

Our findings show the changing perceptions of roles and contributions among participants over time. Patients typically described their role as co-designers in terms simply of sharing their experiences. In contrast, clinicians perceived themselves as co-designers because they were working with patients who were actively involved in decision-making. Levels of engagement were affected by several factors such as time and facilitation, but most participants came to view themselves as co-owners of the intervention. Overall, participants perceived their involvement as a positive experience with clinicians also reporting wider positive impacts on their clinical practice.

## Plain English Summary

Experience-Based Co-Design is a method for helping patients and clinicians work together to improve healthcare services. Studies of participant experiences in projects which use this method and how they perceive the co-designer role are rare. Our study explores how we—patients and clinicians—saw our role and participation as co-designers over time. Our findings are based on our written and verbal reflections of participating in a co-design project aimed at developing an information resource booklet and film for use in cancer care. We also analysed meeting records, email messages between participants and a reflective diary kept by the researcher who was coordinating the project. Our findings show that views of our roles and contributions as co-design participants changed over time. Patients tended to see themselves as ‘co-designers’ simply because they shared their experiences throughout the co-design process. In contrast, clinicians saw themselves as ‘co-designers’ because they were working together with patients and making decisions with them. Factors such as time demands, and the skill of the facilitator affected the commitment of participants to co-design activities. Most participants regard the ownership of the newly developed information resources as being shared. Overall, we viewed our participation in the co-design project as a positive experience with results that will benefit clinical practice.

## Background

Patient and public involvement (PPI) in research is encouraged by research funders, policymakers and research stakeholders [14, 22]. Three broad justifications have been forwarded. Firstly, PPI is often advocated on the grounds that patients have an inherent right to contribute to research concerning their condition and healthcare issues [36]. It is argued that increased accountability and transparency are observed when researchers partner with patients [14]. Related to this, researchers are perceived to have a moral responsibility to acknowledge and pay attention to the voice of individuals who are seldom heard or understood [11]. Secondly, the concept of PPI seeks to address hierarchical power traditionally held by clinicians in clinician-patient relationships [22]. Finally, it is proposed that PPI improves the efficiency of research in terms of recruitment and retention, as well as increasing the quality, relevance and dissemination of results [11, 14].

The use of participatory methods in healthcare improvement has also increased exponentially in recent years [23]; such methods are often applied in the context of the cocreation, coproduction or codesign of health and social care. While PPI is most usually framed in terms of involvement in designing or carrying out research [36], participatory methods in healthcare improvement allow service users to directly engage in coproducing goods and services [23]. Underpinned by democratic value frameworks similar to those sometimes used to justify PPI, participatory methods use creative and reflective tools, methods and processes to support engagement by empowering individuals with lived experience in the process of designing services or interventions to improve health outcomes and experiences [23, 37].

Experience-based co-design (EBCD) is an established standard approach that combines the use of participatory methods and user-design tools and processes to improve health services [25]. It has been used in various healthcare research settings such as mental health [27], acute care [5, 34], and community and primary care [8, 16]. EBCD follows a six-stage sequential process divided into two closely related phases: the (a) exploration and understanding of experiences and (b) co-design [25]. Following study set-up, including initial stakeholder involvement and ethics and regulatory board approvals, the first phase involves observational fieldwork and exploring patient and clinician experiences of a particular service by using a narrative-based approach. Two initial workshops follow with (a) patients only and (b) clinicians only to share reflections and identify improvement priorities. The co-design phase involves clinicians and patients coming together for the first time; at the beginning of an iterative series of workshops patients and clinicians together watch a summary film (touchpoint film) of the patient interviews highlighting key ‘touchpoints’ (moments that shaped overall patient experiences) [1]. Together, through a facilitated process, patients and clinicians then identify their shared key priorities for implementing change. Patients and clinicians then form small co-design groups to actively and collaboratively work on various workstreams that all lead to achieving the set goals of the codesign project. The process culminates in a celebration event where the co-design groups present their work and reflect on their achievements; the next steps of the improvement process are also outlined.

Factors that influence the success of codesign projects in healthcare organisations include having an adaptive, relaxed and inclusive environment [17, 21], skilled facilitation [13, 17], shared understanding of aim and purpose of the process [17], trust and empathy [23] and cultural appropriateness [7]. But there are also reported challenges in the implementation of codesign. It is relatively resource intensive [25] requiring time, money and people; such challenges are often compounded by issues of organisational constraints and limited commitment [4, 10, 17]. Conflict and tension amongst participants have been reported, often due to power issues [4, 10, 12, 15, 21]. Staff participants can view attendance as part of their current role without necessarily seeing themselves as an integral part of the co-design team [4], while patients may struggle with the ambiguity of their roles as service-user and co-designer [17]. Overall, in-depth research on participant experiences and perceptions of their co-designer role—and how these may (or may not) change during a co-design project—is limited.

We intend to share our experiences of participation and involvement in a co-design study that aimed to develop a cancer care intervention. Most of us were unfamiliar with co-design and had differing expectations to begin with, but our involvement has been worthwhile with many learning opportunities along the way. This rewarding experience motivated us to write this reflective paper.

## Aims

This paper aims to explore how participants in an EBCD project came to perceive their role and involvement in co-designing a cancer care intervention over time. It specifically aims to: understand how participants perceived their role as co-designers, describe challenges in implementing the EBCD approach in this project, and explore perceived benefits of involvement among participants.

## Methods

### The main research study

Our findings draw on a doctoral research study that co-designed a theory- and evidence-based behavioural intervention for reducing the effects of chemotherapy-induced peripheral neuropathy (CIPN). CIPN is a side-effect of some chemotherapy drugs used for treating cancer. CIPN symptoms mainly affect the hands, feet or both; symptoms may include numbness, tingling sensations, and pain that can affect an individual’s ability to carry out activities involving their hands and feet [24]. It is important that patients recognise and report their symptoms early so they can be monitored, and their chemotherapy doses reduced, if needed [19]. The literature shows lack of information and support for managing CIPN symptoms [29, 30].

The East Midlands—Leicester South Research Ethics Committee approved the doctoral study (Reference 19/EM/0192). Papers detailing the findings from the main study have been published [31, 32]. The main study was conducted in the cancer centre of a large NHS hospital in London. The study involved observations, semi-structured interviews with patients and clinicians, and a co-design process—including ten co-design workshops attended by patients and clinicians—that lasted for 15 months. Study participants could choose whether to attend only one study activity or more. The patient and public involvement (PPI) group of the study was involved in the full research process and were paid for their time and expertise based on NIHR guidelines [20]. This included developing the study protocol, designing questionnaires, applying for funding, obtaining ethics and research approvals, co-facilitation of co-design workshops, participating in the co-design process, and the analysis and dissemination of findings. MT, an oncology nurse and doctoral student, led the research study and facilitated all the co-design workshops.

**Fig. 1:**
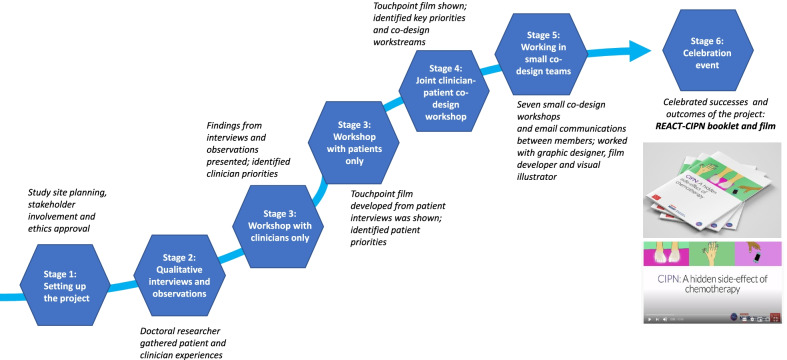
EBCD stages undertaken in the main study

The EBCD process started with study site planning and meetings held by MT with two oncology department leads, one nurse manager and two senior clinicians to obtain project backing for participant and department access, release of staff to attend study activities and support for ethics and regulatory approvals. Figure 1 illustrates the stages of the EBCD process.

### This paper

The findings presented here are based on participants’ reflections, a research diary, email correspondence and meeting fieldnotes. Co-design participants (four clinicians, all four members of the PPI group, one visual illustrator and the doctoral researcher) who attended most of the ten co-design events shared their reflections. Everyone was given the option to either provide a written reflection or discuss their thoughts with MT through a video call. To guide our reflective writing, we were given guiding questions (Table 1).

**Table 1:** Example of guiding questions for reflection

Were your knowledge and experience of CIPN considered?	
Did your level of participation change at all during the whole process? In what way/s?	
Did your beliefs/views/opinions of the co-design process changed when you attended workshops with patients? With clinicians? Joint meetings? In what way/s?	
Were your issues, concerns, views, opinions, voice heard?	
How was the decision-making?	
Were there challenges in the co-design process?	
What facilitated the co-design process?	
Describe your understanding of the term ‘co-design’ before and after the project?	
What was your favourite part of the process? Why?	
Your least favourite part? Why?	
What was it like working with other people with same experience? With clinicians? With the visual illustrator/film developer/graphic designer?	
Do you consider yourself a ‘co-designer’ of the intervention? Why or why not?	
Who owns the intervention?	

MT collected these individual reflections from eight participants by email. JR and CO shared their thoughts through a video call interview with MT using the same guiding questions. These were recorded, transcribed by MT and later reviewed by JR and CO for accuracy. Inductive analysis of reflections was carried out using reflexive thematic analysis [6]. MT coded the individual reflections. Meeting fieldnotes, email correspondence and the researcher’s diary were deductively analysed using the initial themes generated from the inductive analysis of reflections. MT, GR and JA were involved in generating initial themes followed by joint interpretation of the themes. Initial themes were shared with all co-authors for comments and further discussion until consensus was reached. All co-authors agreed on the final themes and reviewed the final version of this paper.

For this paper, the term ‘patient’ is used to include PPI members and one patient participant who has since joined the PPI group, unless specified. Data from field notes, diary and emails were labelled using allocated study identifier numbers of patient and clinician participants in the main study [32].

## Results

Two clinicians, four PPI members, the visual illustrator and the doctoral researcher contributed written reflections ranging from 533 to 1677 words each. In addition, there were two video call interview transcripts; the interviews lasted between 25 and 30 min. Field notes from ten co-design meetings, the researcher’s diary (completed after every co-design meeting) and email correspondence with main study participants between January 2020 and December 2020 were also included in the analysis. The four clinicians comprise an oncology consultant (JR), a chemotherapy nurse consultant (CO), specialist physiotherapist (TW) and specialist occupational therapist (LU).

Five main themes were identified from our analysis: (1) changing perception of roles during the co-design process, (2) defining the term ‘co-designer’, (3) engagement and ownership (4) role of the research facilitator in maintaining momentum, and (5) perceived benefits of involvement.

### Theme 1: changing perception of roles during the co-design process

Information about the co-design process (in this case the EBCD approach) was provided in the participant information sheets, the first of three face-to-face workshops and the first online workshop. Initially, patients felt “*uncertain of what to expect and had no clear idea of how the process would be structured nor the balance of delegation and leading*” (LS). However, patients were motivated by their interest to *“contribute from the patient’s perspective”* (DT*) and “use lived experience to benefit future patients facing the same diagnosis”* (BG) and felt encouraged to participate when they were *“made to feel that what they had to say was going to be useful”* (LB). Although one of the clinicians had been involved in a co-design study (CO) before, most clinicians had no experience of co-design or had not heard of the term before. They felt their lack of understanding of the EBCD process resulted in their unpreparedness, lower engagement with the earlier EBCD stages and unawareness of the time the whole process required.

“I had never really heard the term co-design before and had no experience of working in this way. However, I assumed it meant working with both other professionals and patients closely to develop the project. I assumed there would be meetings in which everyone would share ideas and come to a conclusion about what was needed together. Before this project in hindsight, I don’t think my understanding of this term (co-design) was that in depth.” (LU, clinician)

“I feel that I wasn’t prepared at the start, and wasn’t fully aware that attending all the co design events would be helpful, I also wasn’t sure of the full aim for the project at the start. I remember being invited to an event but I wasn’t aware of the size or background of the project at this point. The design process took up more clinical time than I had thought it would however it is very useful for our patient cohort and I was supported through this by my manager.” (TW, clinician)

Despite lacking clarity on the process and their roles early on, participants highlighted how their understanding of the co-design process grew over time as they went through the stages that *‘allowed everyone to be heard, their experiences captured and commented on before moving on’* (LB, patient participant). The *patient-only workshop* made patients feel an equal part of the group and to begin seeing themselves as co-designers, particularly when they were involved in facilitating elements of the event.

“It was at the patient-only workshop when I really experienced being an equal part of the group of patients as well as doing a little 'facilitation' to help conversations expand on some points. This patient-only workshop was vital in the co-design process as the foundations were laid for us to work in the subsequent stages within groups. It was the patient-only workshop that really started the feeling in me of being one of the 'co-designers’.” (LS, PPI member)

Patients also took some time to decide on who would present the patient-identified priorities at the first joint co-design event with staff; they made sure they chose two representatives who were articulate, gender and age balanced, to ensure their collective views would be heard (Fieldnotes, 27 January 2020).

Both patients and clinicians highlighted the value of the first *joint patient-clinician co-design event* in which they had the opportunity to meet other patients with similar or differing experiences of the condition and work directly with each other; further, clinicians found working with clinical colleagues who were involved in giving information and support to patients with CIPN informative. In this first co-design event, mixed patient-clinician small co-design teams worked together to discuss joint priorities, then reported back to the bigger group. Although all groups nominated a clinician as presenter, it was evident how all members of the groups felt comfortable sharing or adding their views.

‘We thought that our main one, or one of the things that’s flagged up the most by both groups was the patient information side of things but – I don’t know what the rest of the group thinks – but having just heard what Group A said, that’s a really fair point [they all laugh] I’m annoyed I didn’t think of that myself [everyone laughs again] so yeah, [Facilitator: so are you changing…?] oh I don’t feel like I can change without the consent of the group!!! [Laughing] But maybe, no, yeah, so we went with patient information for number one and then we talked about education and training for staff being number two and then we talked about support to manage things following treatment.’ (Meeting fieldnotes, 04 February 2020)

Clinicians acknowledged how perspectives of what was important changed when they worked together and listened to patients’ views at the first co-design event. Below are comments from clinicians following showing of the touchpoint film.

“I think the main sense that we got out of it was that at the start, it was really interesting to hear about the information that patients were given and about the opportunities to absorb the information when they were going through their own side effects. It was the very tip of the iceberg... We feel, as clinicians, sometimes we give you plenty of opportunity and lots of information is always a good thing but when we hear from patients really honestly and very raw data that probably with the mass of information and the way that we deliver it sometimes, you can't digest it in those moments where there is a lot of fear. There is a lot of anxiety in the diagnosis and the information may not cross the threshold in the sense that we think it does as clinicians” (Clinician 05, co-design workshop fieldnotes, 03 February 2020)

“Obviously, we're coming from a different arm. We're very diagnostic. We like to think we're always right but really not because we just watched a video where we're really not right all the time [laughter]. The main issues we really came across was there was a big push around patient information. What are we giving you? How are we giving it to you? Is it digestible? Is it appropriate? All those kinds of questions came up for us as clinicians. There was some discussion around education for staff.” (Clinician 08, co-design workshop fieldnotes, 03 February 2020)

On the other hand, patients felt particularly valued when they worked in the later *smaller co-design groups*. As a PPI member puts it:

“The formation of three small co-design groups, each to focus on one aspect of the outcomes of patient information leaflet, film and CIPN support, was key to ‘intensifying’ the feelings of our experience being valued as well as challenging my skill to evaluate different perspectives to deliver an accurate and wholly acceptable product”. (LS, PPI member)

The *celebration event*, in which the work and results of the *smaller co-design groups* was presented, *‘helped participants appreciate their involvement in shaping the outcomes as well as co-designing the final products’* (LS, PPI member) and*’see the result of all the hard work’* (LU, clinician). All participants expressed that participating in the co-design process was a positive experience. One patient described the phased co-design process as being important:

“We were taken through the different stages in bite-size pieces with constant review built in and with ample opportunity to revise our thoughts and refine what we meant to say. Along the route there was also cross-checking with clinicians and conversation with them about their experiences, and discussions about what they would need going forward. It was fascinating to hear clinicians’ views and to wrangle (good-naturedly) over wording, and over what priorities might be for different stakeholders. I believe this piece of work has been truly co-designed but that it was only possible to be so because the process was rigorously planned and structured.” (LB, patient participant)

### Theme 2: defining a ‘co-designer’

#### Patients

Looking back, most participants considered themselves as co-designers of the intervention. For patients, it was being involved from the very start of the process which meant they saw themselves in this way.

“I do consider myself a co-designer of the intervention, particularly since I was one of the ‘founder members’ of the group, (and very proud to be!) and have taken part in almost every stage of the design, (probably excluding the film-making) over the years it’s taken to bring this to fruition.” (DT, PPI member)

When asked, patients gave exemplars of tasks and contributions to explain why they now saw themselves as co-designers. Most patients mentioned the sharing of their personal experiences to shape the outcomes of the project as key to their role as a co-designer as evidenced in these examples from their reflections:

“Contributed the story of my experience of communicating with clinicians before and during chemotherapy, the effects of CIPN and the emotional and practical impact of both (the effects and the communication) …helped to fine-tune descriptions of symptoms and helped to communicate a patient’s experience of CIPN to clinicians.” (LB, patient participant)

“Shared personal experiences and opinions and worked on a range of different tasks… Got involved with the leaflet illustrations – it’s not easy to express abstract sensations like fuzzy fingers in a drawing.” (DT, PPI member)

“Shared my lived experience of this condition.” (BG, PPI member)

However, data from fieldnotes and email correspondence show patients’ contributions were not only focused on sharing their experiences. Examples of their recommendations, comments and feedback that influenced decisions relating to the intervention development, facilitated learning and enabled collaborative working are presented in Table 2.

**Table 2:** Examples of patient input and recommendations during co-design activities

Recommendations made by patients for the planned intervention during the first co-design workshop (Co-design workshop fieldnotes, 03 February 2020) Small group discussion—Group A Patient 09: I think one would assume they know (Clinician 05: yeah, yeah) and I think one of the big issues with information is their capacity, whether it is inherent or temporary because of their emotions (Clinician: yeah). I think for some people, words won’t do it Patient 09: …I think pictures are a way to go (Clinician 05: yeah) like infographics, just like what MT used, when she explained the process…much more pictures (yeses) Clinician 05: …and for every phase, the time for them, when to deliver. With reviewing these processes, the disparity… it does exist. There are common themes to our agendas, we just have to… Patient 11: …keep going on about it Clinician: yes Patient 09: And also, the information, at the moment we get it the way that I got that…you know, how it might mean…discussing…catching some moments like that… (Patient 11 agreeing to what Patient 09 was saying). Clinician 05: yeah, to go through in your own pace	
Patient suggestions on content of intervention (film) Patient 13: “We are captive while we're having chemo. We're kind of there and so you can find us quite easily. Or even just, it’s an opportunity, as I say, it’s a teaching moment, if you’ve got somebody there and just a small chat, you know… and I know how busy you guys are, I know how understaffed you are…” Clinician 08: “Chemo nurses could come in … you call it a teachable moment [yeah] because they do talk to patients about their symptoms [they do]” (Co-design workshop fieldnotes, 03 February 2020) “I think one film would be sufficient and would hold people’s attention better. People waiting for chemo, if they are like me…find it difficult to focus /concentrate on first chemo. The mind is wandering. Perhaps a viewing with a nurse, someone to answer questions. I realise this is all time consuming. It would only be necessary if the patient showed interest. (Sent by Patient 01 through email, 09 June 2020)	
Patient suggestions on content and format of the intervention (booklet) “I think I agreed with most of what was discussed but wasn’t sure about the “What are the symptoms of CIPN” section. I thought it might be better to separate the five symptoms into 2 areas. With 1 and 3 being the more long-term conditions and 2, 4 and 5 the short-term symptoms. So a heading could say “Symptoms most common during treatment” and then “Longer lasting symptoms”. Also, the worst effect of the drug has not been mentioned which is what can happen if during your infusion you eat or drink something cold. This can cause your throat to close up and it becomes hard to breath. Nurses are well aware of this. It happened to me once and was very frightening. As soon as you have a hot drink though the feeling passes.” (Sent by Patient 13 through email, 23 June 2020) “The general look of the design seems very good with the use of colour blocks a nice way to separate the texts. I would like to comment on the ‘walking or standing for a long time’ section. I thought it was a bit hard to follow and could be rephrased. i.e. – consider sitting whilst waiting for a bus or when having to queue, perhaps taking a folding seat with you. At home a high stool could be useful…I like all the spaces left for the patient to use to ask questions or make comments. I also think the statistic page (2) showing the percentage of people that are affected looks very good” (Sent by Patient 13 through email, 19 October 2020)	

Patients also highlighted what they viewed as *co-designer tasks* during development of the components of the intervention, such as involvement in discussions of content and format, writing, editing and proof-reading, and providing their views on design and production.

“I felt involved in discussions about the format and content of the booklet, so definitely think of myself as a contributor to the project. I also had a role in editing and proofreading.” (BG, PPI member)

“I offered suggestions about wording with the aim of making the intervention as accessible as possible to everyone who uses it.” (LB, patient participant)

“Made sure all communications were easily understood and jargon-free – not as easy as you might think.” (DT, PPI member)

Patients also actively engaged with the designer/illustrator who created visual drawings of CIPN symptoms for the booklet. Their comments showed confidence that the drawings and designs were informed by their experiences:

“I was also fascinated by the conversation with our illustrator who did a good job of visualising the discomforts we described. I am full of respect for her listening skills and commitment to collaborating as there were a lot of opinions to sift on that occasion. Her resulting illustrations answer the brief very well, I think.” (LB, patient participant)

“I can appreciate only too well, how difficult it must be, to illustrate symptoms and related problems, when they are almost entirely invisible and very hard to describe with any accuracy! Even trying to describe to someone else how symptoms affect everyday behaviour, is not an easy task, for those of us living with this condition all the time.” (BG, PPI member)

Two patients did not use the term *co-designer* to label the role they carried out. They were more comfortable using the term *contributor* and may have altered their views or used the term *co-designer* because of how the term was presented to them, as shown in quotes below:

“I probably don’t understand the term ‘co-designer’ and what is implied by it. However, I felt involved in discussions about the format and content of the booklet, so definitely think of myself as a contributor to the project. I also had a role in editing and proofreading if I remember correctly.” (BG, PPI member)

“So, yes, my understanding of the term has altered; previously I would have seen the role I played as that of a contributor. I do, but only because that’s the way it’s been framed. I feel only a slight unease about it because, as I’ve said, the term co-designer infers (to my past self) a more pro-active role than the one I actually played.” (LB, patient participant)

#### Clinicians

Clinicians’ perception of the co-designer role was focused on sharing professional knowledge and advocating for evidence-based information.

“I do consider myself a co-designer. I feel I contributed to elements of the work and process along with lots of others involved. Sharing professional knowledge about assessment and intervention around CIPN, advocating for evidence based best practice advise to be represented, shared personal feelings about layout and language, attending meetings and sharing ideas, and reading through draft versions of resources created and providing feedback”. (LU, clinician)

“I think my professional knowledge and experience was considered throughout the piece especially towards the end when some of my suggestions lead to reworking of the final leaflet. I was also given adequate time to review and provide comments and feedback even towards the end of the project which made me feel as though my opinion was valued.” (TW)

“My knowledge and experience were considered during the discussions about the intervention. I was able to give my advice. Taken on board.” (CO, clinician)

Patients’ increased involvement in decision-making was noticed by clinicians:

“Previously I would have thought co-design was including patients’ feedback in order for clinicians to make decisions, however (in this project) patients have been much more involved in the decision making...I feel like the patients’ voice was very strong.” (TW, clinician)

Although sometimes this needed careful negotiation to ensure the co-design process was informed by the most recent clinical evidence.

“It felt like we had the most participation during the intervention designing stages, during this time there was more call for our input around therapeutic interventions… Main challenge for me was reconciling some elements of how patients felt and what they wanted included, with what we know is advised/evidenced based on a therapy perspective.” (LU, clinician)

**Fig. 2:**
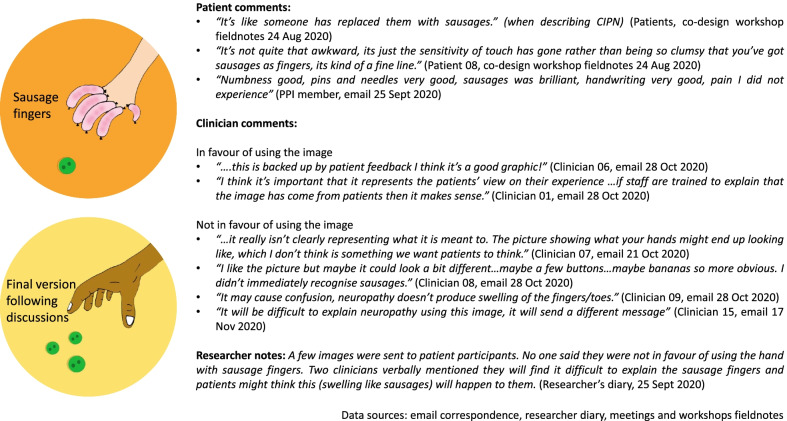
Example of summarised comments during intervention co-design

An example that involved several discussions during co-design involved an image of a hand with fingers that looked like sausages to illustrate *difficulty picking up small objects*. When individuals experience CIPN, they experience numbness, tingling and sometimes motor weakness [28]. These symptoms result in weakened hand grip and less fine movement co-ordination, making it difficult to pick up tiny objects. The image was co-designed by patients with the help of a visual illustrator in one of the workshops. There was conflicting feedback from clinicians and patients; these are summarised on Fig. 2. Initially, patients felt the hand with ‘sausage fingers’ was a good representation based on their personal experience. On the other hand, the clinicians were divided in their views; one group respected patients’ descriptions and experience but the other group were concerned about the risk of misinterpretation. After to-ing and fro-ing, mainly by emails (Fig. 2), the co-design team agreed to change the design to real hands to avoid inaccurate interpretation of the symptoms. A clinician reflected on this specific case:

“There was an image that showed a hand with sausages to show neuropathy. From my point of view, I understood the picture is an outcome of how patients described their symptoms in the study. But if we extrapolate this to a leaflet that is given to a patient who is not aware about what is going on, probably if I put myself in the shoes of the patient, the first thing that I will wonder is if I received this treatment, my fingers are going to swell like sausage...We cannot give this information to the patients because the patients can focus on the worst things and the sausage image can create a lot of misunderstanding. Using real hands is clearer. A picture can be really helpful because sometimes the patients do not read all the texts. However, the picture can send a strong message, so we need to be very careful with these things.” (JR, clinician)

From the viewpoint of the visual illustrator the project was one comprising individuals each with different roles and abilities coming together to achieve the project goal.. Each participant was a ‘co-creator’ of the final output.

“I viewed this project as a collaboration between patients, clinicians and myself, where all of our ideas, experiences, expertise and interpretations were combined to hopefully produce imagery that was striking, representative and engaging. I saw myself as an artist-collaborator on this project, with patients and clinicians also co-creators / collaborators…. As a team, we were all co-creators with different roles and aptitudes, but who could hopefully synergise our energies and strengths to create a powerful final output from the project.” (AW, visual illustrator)

As clinicians and patients carried out their perceived roles in the co-design process, some felt each other’s opinions were challenged, but acknowledged that *‘the group is made up of people from different disciplines with varying viewpoints thus having differences was expected’ (LB, patient participant)*. Some patients ‘*recognised and accepted the views of those with more practical hands-on experience’* (LS and BG, PPI members); patients also acknowledged the opportunity to work with clinicians with one saying they were *‘star-struck’* by the clinicians. For many clinicians, they were open to hearing patients’ views and opinions.

“… a patient’s view on a subject may be different to the professional view - both voices and opinions have their place and are valid and useful in different ways.” (TW, clinician)

Participants also mentioned the merits of the co-design approach for enabling them to work together despite their varying viewpoints.

“I assumed co-design would be more challenging than it was due to the added complexity of lots of people coming from different viewpoints. I have been able to see the value in it from this process.” (LU, clinician)

“Because the process was measured out in steps, the gaps in between meetings allowed time for reflection. I soon understood that participation is as much about holding one’s tongue as it is about being heard and I am grateful for this reminder.” (LB, patient participant)

### Theme 3: engagement and ownership

The lack of protected time, faced particularly by clinicians, led to missed attendance at some co-design workshops. One clinician had to complete tasks over the weekend in their own time. Although some were supported by their line managers and were keen to participate, the effect of the COVID-19 pandemic and their full clinical responsibilities meant that participation was sometimes challenging. This resulted in feeling guilty for not delivering their tasks on time.

“The least enjoyable element over all for me was the difficulty of finding time to put into the research. Wanting to put more time often but not feeling able to. The feeling of constantly being rushed to get things done as often we’d had to leave them until quite last minute or were late. There was a sense of guilt for not being more timely.” (LU, clinician)

“There really isn’t any capacity for me to action it in the next couple of weeks, I’m afraid but we do feel we need to be a part of the working groups to represent therapies” (Email from Clinician 15, 19 February 2020)

This lack of time and busy workload also affected how the research facilitator managed the workshop activities.

“The time was really short but I can see how tired they were. This influenced my facilitation as I did not wish to go overtime. The time of the workshop was suggested by clinicians - afterwork with refreshments and short. I felt guilty when I asked them to do things because they were already very tired.” (Researcher’s diary on clinician workshop, 23 January 2020)

A patient participant also mentioned the phased EBCD approach and the long process –made longer by the pandemic in this project– as impacting negatively on their engagement with the project over time. It also reduced retention and appreciation of the whole process. Nonetheless, there was also recognition of the usefulness of the phased approach to the success of the project.

“The challenges for me were around retaining my appreciation of the ‘whole with its many different parts’, the sequence of those parts and the academic terminology” (LS, PPI member)

“I can’t remember what I thought clearly, but I remember being surprised by the number of stages there were going to be. I had imagined something simpler- a harvest of patients’ experiences and views. On reflection, I realised that would not have been very useful on its own.” (LB, patient participant)

One PPI member, who strongly self-identified as a co-designer, suggested the project funder or doctoral researcher are the owners of the intervention. A patient participant, who did not perceive herself as co-designer, viewed the doctoral researcher as the owner because it was the doctoral researcher’s original idea. In contrast, most participants (all clinicians and two PPI members) felt everyone ‘owned’ the intervention because they all contributed to the co-design process. Excerpts of participant reflections about ownership are presented in Table 3.

**Table 3:** Participant reflections about ownership of the co-designed intervention

Patients	Clinicians
“I presume all members of the collaborative group technically own it (the intervention), because we all contributed to producing it.” (BG, PPI member) “I can't name us all as individuals, but I feel the 'ownership' is no one person; it is definitely a 'Whole Group Ownership'. We were definitely led by MT, guided by MT but the intervention outcomes are a wholly collaborative product, an example of 'Gestalt', i.e. the whole is greater than the sum of the individual parts.” (LS, PPI member) “I’m confident that I don’t own it. MT owns it because she conceived the project and drove it through to fruition.” (LB, patient participant) “I would say that you (MT) own the intervention, since it was your original idea and we simply helped you develop it into its final form…I suppose there’s a case for saying the funder is the owner, having ‘bought’ it, so to speak, and you are the custodian.” (DT, PPI member)	“I feel everyone involved owns the intervention. However, I do feel that the researcher(s) were really key to the project as they facilitated the movement and progress and did the bulk of putting work together and amending as required. They also were fundamental in ensuring ideas were pulled together throughout as due to the collaborative design there were lots of different ideas over a broad range of areas, so I feel they hold a lot of the ownership.” (LU, clinician) “I think everyone who has been involved in the process owns the intervention. This will benefit not only the patients but also the people who look after them because at the end of the day we want the best for their patients and the patients want the best for themselves.” (JR, clinician) “The patient owns the intervention. I suppose the correct answer would be the patients, because it's…Well…I'm just thinking now. They should be Co-owned. I suppose there is the difference between ownership and responsibility. To keep it moving forward, it is important to have a person that looks after it if you like and maintains it. But it's owned by the patients and staff who developed it.” (CO, clinician)
Researcher “At the start, I always had to remind co-design participants this was not just my project but it was also theirs and that their views and decisions mattered and will be considered…Co-design members seemed to be more relaxed when giving their views during the later stages; I felt it was no longer my project but theirs. I think this was the reason why towards the end, it was much easier to obtain their views and decisions…At the start, there was a constant use of ‘us and them’ among co-design participants, this was changed to ‘we’ at the completion of this phase.” (MT, doctoral researcher)	

The doctoral researcher’s reflections showed evidence of patients and clinicians taking more ownership by using the word ‘we’ (as opposed to ‘us’ and ‘them’) over time and the growing ease with which they gave their opinions and decisions in the latter stages of the co-design process. But patients and clinicians together bestowed a different type of ownership on the doctoral researcher – as custodian – for keeping the project moving forward, facilitating the co-design process, guiding the group and being the named individual who sustained the intervention moving forward.

### Theme 4: role of the research facilitator in maintaining momentum

Participants commented on factors that helped sustain their engagement in the co-design process. These included meeting face-to-face and the virtual meetings when pandemic restrictions came into effect. They also highlighted the perceived mutual respect between participants and being made to feel useful or listened as motivating them to continue to participate. Participants also commented on the role of the research facilitator –in this study, the doctoral researcher– as essential to keeping the momentum going.

“You need a really good facilitator if you like to bring everyone together and to hear everyone's ideas. So that people do not sort of dominate the conversation and you get everyone's expertise and experience.” (CO, clinician)

“Patients' opinions were valued equally and MT offered us a range of ways to give them.” (LB, patient participant)

“I do feel that the researcher(s) were really key to the project as they facilitated the movement and progress and did the bulk of putting work together and amending as required. They also were fundamental in ensuring ideas were pulled together throughout as due to the collaborative design there were lots of different ideas over a broad range of areas.” (LU, clinician)

Many also highlighted the essential role of the doctoral researcher in bringing views, ideas and decisions together. Participants also identified tasks and skills of the research facilitator that supported or encouraged their participation and involvement, particularly when the co-design process was impacted by pandemic restrictions. These are illustrated in Table 4.

**Table 4:** Tasks and skills of the research facilitator that supported participant involvement

	Identified by patients	Identified by clinicians
Skills	Skills in using virtual platforms Communication and updates Used different participative techniques	Pulled the information together and brought together views
Attributes	Confidence as a leader Coordinator of people, physical resources Problem-solving approaches	Sensitivity
Approaches	Allowed time for reflections Valued opinions equally/placed premium value on experiences Offered a range of ways for giving opinions Took through process where everyone was allowed to be heard, experience captured and commented on before moving on Created a sense of equality, inclusiveness and appreciation for each other's contribution; vital for enabling to work as a team Nourished positive feelings	Made participants feel their perceptions were valued and suggestions taken seriously Provided time allowances to include late contributions

A participant highlighted that the trusting relationship with the research facilitator helped them to feel safe when sharing their experiences of cancer and treatment side-effects:

“I became increasingly confident to share detail of my cancer and its effects, something I now realise I have not chosen to do in any other relationship. This tells me, the research facilitator enabled me to feel 'safe' with a part of my life I prefer to keep 'quiet' as much as possible so as not to become ‘a victim’ of cancer....just be a person who happens to have this experience within their life.” (LS, PPI member)

For the researcher, having overall responsibility for running the project involved taking on many roles that required different forms of delegated and presumed power –from the co-design team which enabled her to sustain interest and momentum.

“Upon reflection, some power (as enabler) also rests on me as co-ordinator/researcher because I made sure everyone was heard and made opportunities for this to happen even when communication and working together was made difficult by the pandemic…During this process, I wore so many hats such as co-ordinator, navigator, note-taker, mediator, doctoral researcher, but my nurse hat has always been in the background.” (MT, doctoral researcher)

### Theme 5: perceived benefits of involvement

Participants perceived their participation was beneficial and contributed to the co-design of the intervention. Patients felt they added credibility by sharing their experiences and contributed to the comprehensibility and relevance for patients and clinicians who may utilise the intervention in the future. Similarly, clinicians felt their perspectives, opinions, knowledge and experiences were well considered and shaped the outcomes of this co-design project.

“I became part of an expanding group of people that shared experiences, co-designing the intervention along with interested healthcare professionals, adding credibility to the research right from the start, making sure the project started off on the right footing and, probably most importantly, helping to make the research, and the resulting ways to communicate the intervention, relevant to patients, their families and everyone who contributes to their treatment, including clinicians from a range of specialties.” (DT, PPI member)

“Our perspectives and opinions as professionals felt valued and considered at all stages. It felt we were sought out for our input which was validating.” (LU, clinician)

“I think my professional knowledge and experience was considered throughout the piece especially towards the end when some of my suggestions lead to reworking of the final leaflet. I was also given adequate time to review and provide comments and feedback even towards the end of the project which made me feel as though my opinion was valued.” (TW, clinician)

“My knowledge and experience were considered during the discussions about the intervention. I was able to give my advice…You can really tell it's being developed that the booklet and film were developed through in-depth research because they have a very high quality. Very carefully thought through, patient-centred and patient-focused. It's what patients will find useful. I think it will be really good for teaching as well for staff.” (CO, clinician)

Patients found participation beneficial because they were able to meet other people and discuss their shared experiences, as well as contributing by using their patient voice during the co-design process.

”I found the experience of working with a group of patients and clinicians on a joint co-design project, to be positive and worthwhile. It was most interesting to meet other people with experience of CIPN, and to find similarities and differences in how the condition has affected us individually.” (BG, PPI member)

“When I found out online about a research project that was planning to design an intervention for CIPN, I signed up immediately (with my fuzzy fingers), because I was really interested in contributing from the patient’s perspective.” (DT, PPI member)

On the other hand, clinicians mentioned wider impacts of participation on their job roles and practice. The process enabled them to see the effect of CIPN symptoms from different perspectives.

“I found it a very positive experience. Both for the outcome that we managed to achieve but also personally and from a professional point of view, because it has enabled us to experience a different way of designing something and working using co-design with patients as well. The conversations and the experiences given by patients have enabled us to gain greater insight into what actually and personally impacts the symptoms and I always think it gives us a different perspective when it is presented from a patient rather than being just in a consultation when we are probing and asking although we try to make sure that our treatments are patient-led in conversation.” (Clinician 07, Meeting fieldnotes 07 December 2020)

Their participation and working together enabled them to network with other colleagues and promote their services to those who they do not closely work with.

“It was very useful chance to get to know other people, network and promote services. Developing relationships with professionals you don’t necessarily see a lot is hard so this was a really useful opportunity. It seemed to highlight that there is need across services to promote where to signpost patients with this symptom to for support.” (LU, clinician)

“I think everyone sees neuropathy in a different way and this is very important to take on board for us in the daily work. I don't know what others’ opinion of the doctors. I think it is very important to be in one room talking about neuropathy because we do not hear each other’s views all the time, with nurses or the physios, we do not work together all the time.” (JR, clinician)

## Discussion

Previous research shows that shared understanding among participants about the objectives of co-design processes helps with participation [17]. Co-design permits users of the service –in this project, the patients– to become active participants in the design team as “co-designers” and as the experts about their own experiences [35]. Our findings highlight that even if lacking an understanding of the co-design process at its beginning, we were primarily motivated to take part by our interest to improve services and patient experience. Whilst provided with information about EBCD at various stages, it was our actual experience of going through the stages and engaging in the process that improved our understanding and strengthened feelings of being valued and perceiving ourselves as co-designers. Researchers using the EBCD approach should find innovative ways to support perception and understanding of the EBCD process, stages and roles early on to enhance the experience of co-design participants.

EBCD promotes partnership and collaborative working between patients and clinicians, in which patients are included as active partners in change [25]. The co-design process provided opportunity for us—clinicians and patients—to share experiences which led to change in how we viewed priorities, improved our understanding of the problem and increased our shared commitment to implement change. Similar to an earlier study, the joint event enabled mutual understanding which narrowed the gap between patients and clinicians through hearing each other’s viewpoints [8].

The creative exchange between (a) patients, processes, systems and spaces and (b) the people who deliver a service builds insights into patients’ ideas, motivations and needs [33]. Clinicians in our study thought they were co-designers because they were working with patients who were actively involved in making decisions. Although there were instances in which negotiations had to be undertaken, the transition to sharing responsibility and control was not an issue for clinicians. On the other hand, patient participants considered themselves as co-designers primarily because they shared their experiences but not because they worked in partnership and collaboration with clinicians in developing the CIPN intervention. However, data from the fieldwork showed that patients actively put forward suggestions for change that were acknowledged by clinicians and discussed in the group; many were subsequently included in the intervention prototype.

Results of an earlier service co-design study suggested participants felt they were going through a process of consultation rather than being involved in co-designing (despite being involved in co-design activities) [4]. There may be several explanations as to why in our study, patients’ perception or description of the co-designer role did not reflect the actual roles they performed. For example, patients frequently based their suggestions on their personal narratives and actual experience of the service; the uniqueness of their contribution through their story and memories of their experiences were probably easier to recall. In EBCD, the direct engagement of patients and clinicians in the narratives and stories enables joint analysis and evaluation to facilitate the change process [1, 34]. However, the patients in our study did not seem to fully realise how their experiences facilitated learning for everyone—particularly the clinicians—and how their viewpoints affected group decisions, dynamics and overall direction of the co-design project [8]. Further, some patients felt somewhat overawed by clinicians’ experiences and expertise; they may have thought they had less to contribute than their clinician counterparts [17]. Finally, half of the co-design workshops occurred virtually due to pandemic restrictions—followed by email communications—which limited our personal interactions and may have made the process feel less like a collaborative undertaking as compared to earlier stages of the study.

There were factors that made participation and engagement in the co-design activities difficult for patients and clinicians. Echoing findings of previous studies, the lack of dedicated time for involvement—despite having support from their managers—was an issue for clinician participants [2, 4, 10] although the pandemic may have amplified this challenge. Chisholm et al. [8] recommend that EBCD has to be acknowledged and fit with institutional and personal values to achieve successful engagement, genuine commitment and motivation to engage in the process. Co-design processes should be tailored to enable genuine participation,careful selection of co-design methods and tools should be applied to fit the needs of the project [3]. Stakeholder commitment and buy-in influence effective implementation of any project. Hence, obtaining consistent support from senior management and decision-makers is vital to ensure full engagement, sustainability and promoting integrity of the project [8, 10].

We noted the length of the co-design process as one of the factors that affected participant engagement and full appreciation of the process; this resonates with findings from other co-design studies [4, 8, 10]. We all highlighted the enabling role of the research lead/facilitator in encouraging participation despite practical disruptions due to the pandemic and resource constraints. Many previous studies have established the fundamental importance of high quality facilitation and project leadership for the success of the EBCD approach [9, 17, 18], particularly for moving things forward during early phases of group formation and integration [8]. It was also evident that regular updates and summaries from the research lead/facilitator were useful mechanisms for ensuring continuity and our engagement.

Another important consideration for co-design is the concept of ownership [21]. Most of us who contributed to this paper perceived ourselves as co-owners of the intervention; we participated in most of the EBCD stages. Interestingly, clinicians readily acknowledged co-ownership of the EBCD intervention whilst some patients were more reserved in calling themselves co-owners. It was worth noting that despite this sense of ownership by co-design participants, we particularly identified the need for a person to take primary responsibility or be the custodian for the intervention. In this study, we identified the doctoral researcher as taking this responsibility although two lead clinicians and some PPI members were still actively engaged for the next implementation and evaluation phase of the wider study. It is essential that EBCD projects progress from the active co-design phase into more formal testing and implementation [21].

### Limitations

Our study is not without limitations. Firstly, the co-design process occurred at the start and through the height of the pandemic, which may have affected the timeframe and our experiences, engagement and commitment. The flexible nature of EBCD allowed participants to join even if they were unable to attend earlier stages or were able to resume their participation following disruptions caused by pandemic restrictions or personal reasons. Nonetheless, some participants were unable to re-engage as we headed towards completion of the process. Secondly, our reflections of the process were collected several months following the active co-design phase and were susceptible to recall bias. To mitigate this, we used other sources of data e.g. meeting fieldnotes, researchers’ diary and email correspondence in our analysis to validate themes that emerged from the analysis of our collective reflections. We recommend future co-design studies explore participants’ experiences simultaneously with the main study to capture real-time experience data. Thirdly, those of us who shared our retrospective reflections of the process were those who attended most of the ten co-design events. Those who were only able to engage in one or some workshops may have different perspectives from us who have highly engaged in the codesign process. Whilst this paper focuses on our experiences as applied health service researchers and study participants of the use of EBCD in developing a complex health intervention, our findings may also contribute to discussions of the potential role of co-design in other disciplines such as public administration [26].

## Conclusion

Our findings show changing perceptions of a co-design process and the roles of patients and clinicians over time. Patients described their co-designer role in terms of their sharing of experiences while clinicians perceived themselves as co-designers because they were working with patients who were actively involved in decision-making. Levels of engagement were affected by several factors such as time and facilitation; most of us viewed ourselves as co-owners of the co-designed intervention. Overall, we perceived our involvement as a positive experience with clinicians reporting wider positive impacts on their clinical practice.

## Data Availability

The datasets generated and/or analysed during the current study are not publicly available due to privacy and confidentiality agreements but are available from the corresponding author on reasonable request.

## Abbreviations

CIPN: Chemotherapy-induced peripheral neuropathy
EBCD: Experience-Based Co-Design
PPI: Patient and Public Involvement
